# Rigorous Accounting for Dependent Scattering in Thick and Concentrated Nanoemulsions

**DOI:** 10.1021/acs.jpcc.3c08072

**Published:** 2024-04-08

**Authors:** Ricardo Martinez, Abhinav Bhanawat, Refet Ali Yalçin, Laurent Pilon

**Affiliations:** † Mechanical and Aerospace Engineering Department, Henry Samueli School of Engineering and Applied Science, University of California, 420 Westwood Plaza, Los Angeles, California 90095, United States; ‡ California NanoSystems Institute, University of California, Los Angeles, California 90095, United States; § Institute of the Environment and Sustainability, University of California, Los Angeles, California 90095, United States; ∥ Saint-Gobain Research Paris, Aubervilliers 93300, France

## Abstract

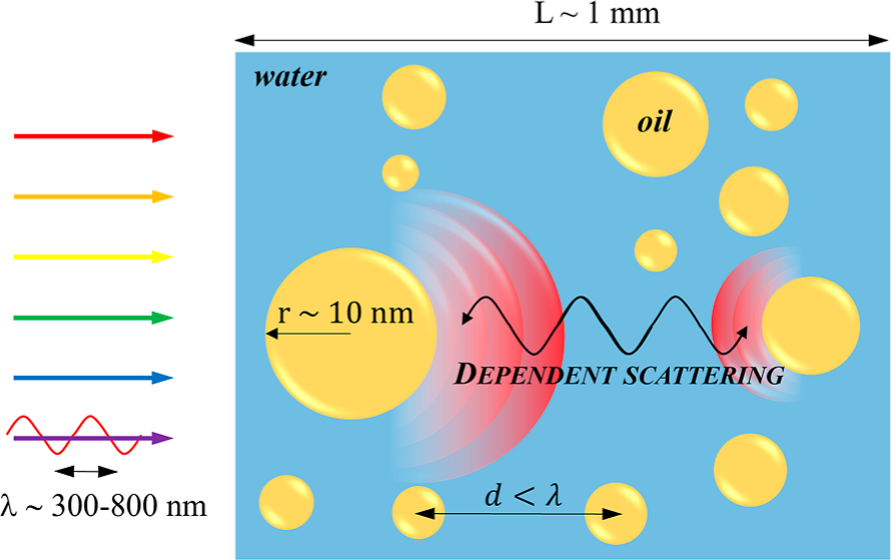

Concentrated and thick oil-in-water nanoemulsions have been observed to become more transparent with increasing oil volume fraction. This study demonstrates rigorously experimentally and numerically that such unusual behavior is due to dependent scattering including not only far-field but also near-field effects. Indeed, when the droplet concentration is sufficiently large, their interparticle distance becomes small compared to the wavelength of light and scattering by a given droplet may be affected by the proximity of others. This situation is referred to as dependent scattering. Light transfer through nanoemulsions and other colloids has previously been modeled by solving the radiative transfer equation accounting for dependent scattering using the static structure factor based on far-field approximations. Here, oil-in-water nanoemulsions were prepared with oil volume fraction ranging between 1 and 20% and a peak droplet radius of 16 nm. The spectral normal–hemispherical transmittance of the different nanoemulsions in 10 mm thick cuvettes was measured experimentally between 400 and 900 nm. Numerical predictions for nonoverlapping randomly distributed nanoscale oil droplets in water and accounting for dependent scattering including near-field effects—using the recently developed radiative transfer with reciprocal transactions (R^2^T^2^) method—were in excellent agreement with experimental measurements. Simulations revealed that assuming independent scattering underestimated the normal–hemispherical transmittance even for a relatively small oil volume fraction. Additionally, simulations using the dense medium radiative transfer (DMRT) and static structure factor predicted that dependent scattering prevailed for oil volume fractions slightly greater than those predicted by the R^2^T^2^ method. Interestingly, the DMRT method predicted large increases in transmittance when the oil droplet size and volume fraction were larger than 10 nm and 10%, respectively. Finally, simulations also revealed that dependent scattering enables the design of oil-in-water nanoemulsions to backscatter or absorb light by tuning the oil droplet size and volume fraction. The results validate that the R^2^T^2^ method could be used to characterize nanoemulsions or to investigate their formation, composition, and stability for drug delivery, food, and cosmetics applications. Future studies could extend the use of the R^2^T^2^ method to colloidal suspensions with particles of arbitrary shapes and to radiation transfer of polarized light in turbid media.

## 1 Introduction

Nanoemulsions are metastable mixtures of two immiscible fluids in which the dispersed phase consists of droplets with a diameter smaller than 100 nm.^1^ Such small droplets can be obtained by applying extreme shear stress to emulsions so as to rupture their micron to millimeter size droplets.^2^ In fact, this process changes a turbid microemulsion into a nearly transparent nanoemulsion, while the volume fraction of oil in water remains unchanged. Nanoemulsions have been studied extensively due to their superior stability.^2−5^ For example, they have been investigated for drug delivery^6,7^ and food preservation^6,7^ thanks to their abilities to encapsulate bioactive and antimicrobial compounds.^7−9^ Their small droplet size, texture, and optical clarity have also made them ideal vehicles for delivering active ingredients in cosmetics.^7^ In addition, nanoemulsions have shown promises in the oil and gas industry as potential agents for heavy oil recovery.^10,11^ In all these different applications, accurate characterization of nanoemulsions is important.^6,9,12^ Optical techniques, such as dynamic light scattering (DLS), have been utilized to measure the size distribution of oil droplets in nanoemulsions.^2,9,12−14^ However, in thick and/or concentrated nanoemulsions, multiple and/or dependent scattering may occur. In fact, they become more transparent as the oil volume fraction increases.^13^ Previously, researchers have noted the larger transmittance^15^ and related it to the effect of osmotic compressibility on the structure factor.^16,17^ The structure factor has been used to explain qualitatively the reduction in turbidity, *i.e.*, the increase in transmittance in colloids with increasing volume fraction.^18−22^ However, the structure factor correction accounts for dependent scattering only by using a far-field approximation to modify the independent scattering solution of the scattering coefficient but does not account for near-field effects.^18−21,23−25^ However, the latter can affect the appearance of nanoemulsions, which is an important consideration in food and cosmetics applications, for example.

The objective of this study is to explain and predict the unusual optical transparency observed in thick and concentrated nanoemulsions.^13^ Oil-in-water nanoemulsions were prepared and characterized experimentally for a wide range of volume fractions. Measurements of the spectral normal–hemispherical transmittance of the different nanoemulsions in the visible and near-IR regions were compared with predictions by different analytical or numerical methods to assess the importance of scattering and absorption phenomena and the effects of droplet size and volume fraction on the optical properties of nanoemulsions.

## 2 Background

### 2.1 Radiative Transfer Equation

The steady-state radiative transfer equation (RTE) governing the spectral radiation intensity  (in W/m^2^μm sr) in any arbitrary direction  in an absorbing, scattering, and nonemitting suspension can be expressed as^26^
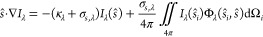
1where κ_λ_ and σ_s,λ_ are the effective absorption and scattering coefficients (in m^–1^) of the heterogeneous medium, respectively. The scattering phase function  represents the probability that the incident radiation from an arbitrary direction  can be scattered in direction . The first term on the right-hand side of eq 1 accounts for the attenuation of the light intensity by both scattering and absorption along direction , while the second term accounts for multiple scattering. If the radiation experiences at most one scattering event as it travels through the suspension, the multiple scattering term can be ignored.

In the absence of multiple scattering and boundary reflections, the solution of the one-dimensional RTE across a suspension of thickness *L* (in m) can then be expressed in terms of the normal–normal transmittance *T*_nn,λ_ as^26^
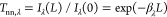
2where β_λ_ = κ_λ_ + σ_s,λ_ is the extinction coefficient (in m^–1^). Equation 2 is known as the Beer–Lambert law^27^ and has been used to determine experimentally the extinction coefficient of nanoemulsions and of colloidal suspensions from normal–normal transmittance measurements.^13,28,29^ However, the Beer–Lambert law is not valid in situations when multiple scattering and/or boundary reflections occur.^26^

### 2.2 Independent Scattering

Independent scattering refers to the situation when the scattering coefficient σ_s,λ_ (in m^–1^) of a two-phase suspension can be estimated from the superposition of contributions of individual scatterers. Such an assumption was shown to be valid when the minimum distance *d* between adjacent scatterers in a suspension is more than 5 times larger than the wavelength λ of the incident radiation.^30^ Then, the scattering coefficient σ_s,λ_ can be expressed as the sum of the scattering cross-sections of all scatterers divided by the volume of the suspension. For emulsions with monodisperse spherical droplets of radius *r*_s_, the effective scattering coefficient σ_s,λ_ and absorption coefficient κ_λ_ can be expressed as^23,26^
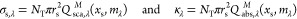
3where *N*_T_ = 3*f*_v_/4π*r*_s_^3^ is the number density of droplets in the suspension (in #/m^3^) and *Q*_sca,λ_^*M*^(*x*_s_,*m*_λ_) and *Q*_abs,λ_^*M*^(*x*_s_,*m*_λ_) are, respectively, the scattering and absorption efficiency factors of an individual spherical droplet obtained from the Lorenz–Mie theory. Here, *x*_s_ = 2π*r*_s_/λ is the droplet size parameter and *m*_λ_ = *m*_s,λ_/*n*_m,λ_ is the droplet relative complex index of refraction defined as the ratio of the complex index *m*_s,λ_ = *n*_s,λ_ + *ik*_s,λ_ of the droplets to the refractive index *n*_m,λ_ of the nonabsorbing surrounding phase. Moreover, the effective scattering phase function of the suspension may be expressed as that of a single oil droplet, *i.e.*, .^26^

In general, particle suspensions or nanoemulsions feature polydisperse particles or droplets with size distribution *f*(*r*_s_).^26^ Then, their effective scattering and absorption coefficients assuming independent scattering are expressed as^26^
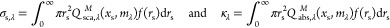
4

Similarly, the effective scattering phase function of the suspension is given by^26^
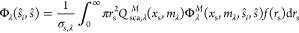
5

### 2.3 Dependent Scattering

Dependent scattering refers to the situation when the interparticle distance *d* is of the same order of magnitude as that of the wavelength λ. This corresponds to the case when the particle suspensions or nanoemulsions are sufficiently concentrated. Then, adjacent particles alter the electromagnetic wave incident on a given particle, and the superposition principle no longer applies. Then, far-field and near-field effects must be accounted for, and the scattering and absorption coefficients of concentrated suspensions must be determined by solving Maxwell’s equations through one or more representative ensembles of particles.^31^ However, such an approach (*e.g.*, the T-matrix method) is computationally intensive and cannot be used, in practice, for highly concentrated suspensions with thicknesses much greater than the wavelength λ.^23^

Alternatively, far-field approximations have been proposed to account for dependent scattering. For example, Mishchenko^18,19^ utilized the static structure factor as a correction in the asymmetry factor and scattering coefficient of soil particles and planetary regoliths. Though the static structure factor does not account for near-field effects and does not alter the absorption cross-section calculated by the Lorenz–Mie theory, it has been widely used to account for dependent scattering included in colloidal suspensions.^20−22^

Similarly, Tsang and Ishimaru^32^ developed the dense medium radiative transfer (DMRT) theory based on the second moment equations of the electromagnetic wave theory. The authors separated the scattered field into coherent and incoherent components. The incoherent component satisfied the Bethe–Salpeter equation, and the quasi-crystalline approximation was used. Thus, the DMRT method solves the RTE by quasi-crystalline approximation on the ensemble-averaged Mueller matrix and by disregarding the coherent backscattering phenomenon.

Recently, Muinonen *et al.*(24) and Väisänen *et al.*(25) developed the radiative transfer with reciprocal transactions (R^2^T^2^) method based on the Monte Carlo method for studying radiation transfer in astrophysics. Here, the authors accounted for dependent scattering rigorously by including near-field and far-field effects. In brief, the method consists of three steps described in detail in ref (23). First, *N* ∼ 300–900 spherical particle ensembles of randomly distributed particles are generated such that the simulated domain is ergodic. Then, the incoherent T-matrices of the *N* particle ensembles and their incoherent scattering coefficient  and absorption coefficient  are computed using the superposition T-matrix method. Lastly, the Monte Carlo ray tracing method is used to solve statistically the RTE using the particle ensemble-averaged incoherent extinction coefficient defined as
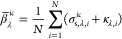
6and the randomly sampled scattering albedo of ensemble “*i*” given by
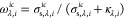
7

Similarly, the polarization-dependent scattering matrix is generated at each scattering event using a randomly sampled incoherent T-matrix and local spherical wave function coefficients randomly sampled for one of the *N* particle ensembles.^23^ Contrary to classical RTE solvers, which consider only plane waves as the incident waves to determine the radiation characteristics of the suspensions, the R^2^T^2^ method accounts for the nonplanar nature of the scattered waves and determines the direction of a wave incident on a particle ensemble from the T-matrix calculated for the previous particle ensembles considered. As such, the method requires us to perform wave tracing as opposed to ray tracing and is, thus, more computationally intensive. Moreover, averaging the radiation characteristics of a large number of particle ensembles or calculating the radiation characteristics of a sufficiently large and “representative” volume of the nanoemulsion to plug into conventional Monte Carlo simulations is conceptually very different from the R^2^T^2^ method and would not give the same results. Indeed, this approach would neither correct for coherent scattering at the volume boundary or account for the nonplanar nature of the scattered waves within the medium. Note that fluctuations in the seemingly random particle spatial distribution in the different particle ensembles and in the associated scattering and absorption coefficients capture the actual thermal agitation of the droplets in the nanoemulsions.

### 2.4 Light Transfer through Colloidal Suspensions

Numerous studies have considered light transfer in colloidal suspensions. McNeil and French^33^ experimentally measured the spectral diffuse reflectance *R*_λ_ and transmittance *T*_λ_ of titania nanoparticles of radius *r*_s_ = 100 nm and volume fraction *f*_v_ ranging between 1 and 3% suspended in an optically clear resin matrix of thickness *L* varying from 12 to 16 μm within the spectral window of 280–850 nm. The authors then determined the system’s effective scattering and absorption coefficients and scattering phase function by relating them to the Kubelka–Munk parameters and solving the inverse problem. The authors compared their results of the above radiation characteristics to that predicted by the Lorenz–Mie theory and noted deviations at wavelengths above 400 nm attributed to dependent scattering effects. They speculated that dependent scattering reduces the backscattering of the suspension. However, the authors did not attempt to explicitly account for dependent scattering in their numerical predictions of diffuse reflectance.

Werdehausen et al.^34^ used the T-matrix method to predict numerically the transmittance and reflectance at a wavelength λ = 800 nm of a few microns thick PMMA slab containing monodisperse ZrO_2_ nanoparticles with radius *r*_s_ ranging from 4 to 80 nm and volume fraction *f*_v_ spanning 1 to 30% to study the system’s transition from bulk homogeneous to heterogeneous media. They successfully showed that the suspensions could be treated as a homogeneous medium with some effective refractive index given by the so-called Maxwell–Garnett–Mie theory when the nanoparticles had radius *r*_s_ < λ/200 for all volume fractions considered. Additionally, their results also revealed that the effective medium approximation failed to accurately predict the complex part of the effective complex index of refraction, especially at high volume fractions.

Many studies have analyzed the effect of the structure factor of the optical behavior of colloidal suspensions.^20−22^ For example, Wang and Zhao^20^ studied numerically the effect of the structure factor on the scattering coefficient and asymmetry factor of charged colloidal suspensions of TiO_2_ or polystyrene nanoparticles in water with volume fraction *f*_v_ ranging between 1 and 30%. The authors used the Yukawa hard sphere formulation^35^ to determine the correlated positions of each scatterer in the medium and the corresponding static structure factor. They controlled the shape of the structure by introducing salt in concentrations ranging from 0.1 to 10 mM, which in turn altered the interaction among titania particles. The authors observed a decrease in the scattering coefficient σ_s,λ_ and asymmetry factor as the salt concentration increased. They concluded that alterations to the electric and magnetic dipole excitations caused by the structural correlations can have a strong effect on the scattering properties of the suspension. It is important to note that this study was purely numerical and did not validate their predictions against experimental measurements.

Recently, Yalçin *et al.*(23) extended a computational framework based on the R^2^T^2^ method^24,25^ to simulate light transfer through thick plane-parallel slabs of concentrated colloidal suspensions accounting for dependent and multiple scattering including near-field effects. This method consists of three steps, described previously in detail in ref (23). Yalçin *et al.*(23) validated their computational framework by successfully comparing its predictions of the normal–hemispherical transmittance *T*_nh,λ_ of colloidal thin films with those obtained by solving Maxwell’s equations using the numerically exact T-matrix method or the DMRT method.^32^ Their computational framework was also validated against experimental measurements of *T*_nh,λ_ for colloidal suspensions of polydisperse silica nanoparticles in water with radius *r*_s_ ranging from 5 to 15 nm and volume fraction *f*_v_ ranging between 2 and 15%. In the same study, the authors determined that dependent scattering prevailed in nonabsorbing colloidal suspensions with volume fraction *f*_v_ > 2%, the particle size parameter *x*_s_ < 0.6, and 1.2 ≤ *m*_p_/*n*_m_ ≤ 2.5.

### 2.5 Light Transfer through Nanoemulsions

Few studies have investigated light transfer through nanoemulsions.^13,28,36^ Graves and Mason^13^ experimentally measured the normal–normal transmittance *T*_nn,λ_ of various fractionated PDMS oil-in-water nanoemulsions with the mean droplet radius  = 32, 47, and 89 nm as functions of wavelength λ ranging between 250 and 800 nm for oil volume fraction *f*_v_ ranging between 1 and 45% in cuvettes of path length *L* = 0.1, 0.5, and 1 mm. The transmittance *T*_nn,λ_ was found to decrease with increasing volume fraction *f*_v_ up to about *f*_v_ = 10–15% (see Figure 1 in ref (13)). Surprisingly, *T*_nn,λ_ started increasing with increasing volume fraction *f*_v_ above 15%. Unfortunately, no physical explanation was provided. In addition, the authors retrieved the nanoemulsion’s extinction coefficient β_λ_ (in m^–1^) by using Beer–Lambert law and the spectral normal–normal transmittance for the thinnest cuvette path length to ensure single scattering. The data were fitted to the *ad hoc* expression given by
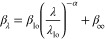
8

**Figure 1:**
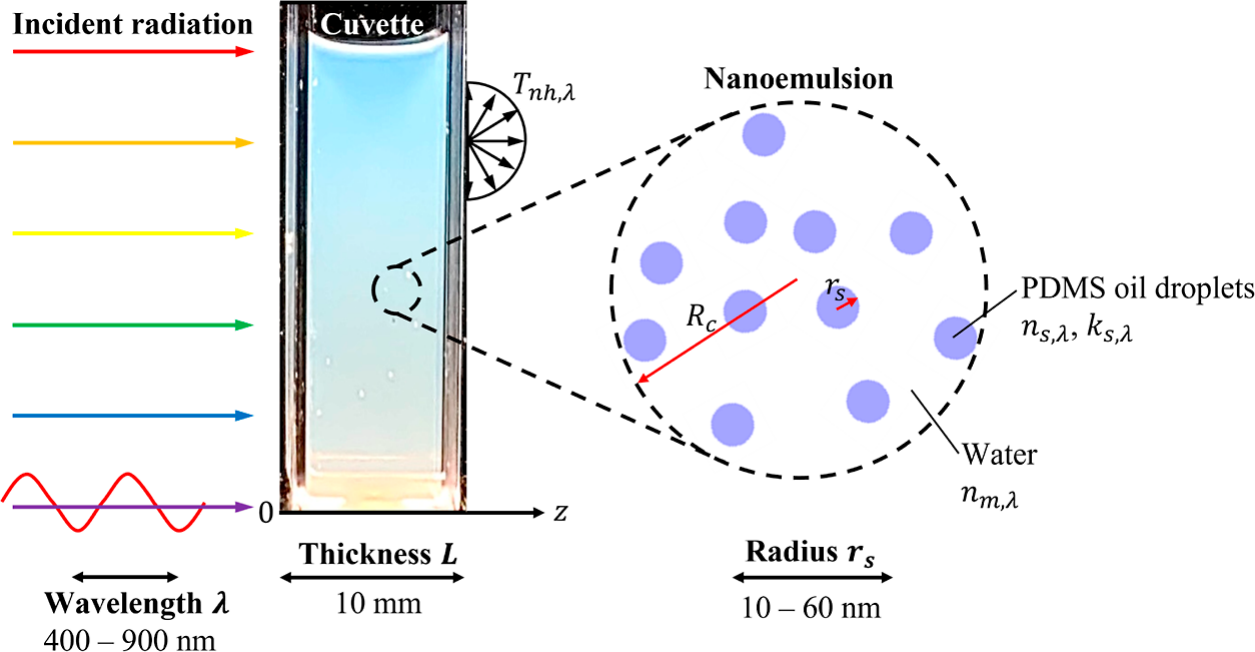
Schematic of a PDMS oil-in-water nanoemulsion in a cuvette of path length *L* subject to collimated and normally incident radiation of wavelength λ. The nanoemulsion consisted of polydisperse oil droplets of size distribution *f*(*r*_s_), complex refractive index *m*_s,λ_ = *n*_s,λ_ + *ik*_s,λ_, and volume fraction *f*_v_ randomly distributed in water of refractive index *n*_m,λ_.

Here, the fitting parameter β_lo_ was the extinction coefficient measured at wavelength λ_lo_ = 250 nm and β_∞_ accounted for residual scattering at high wavelengths. The authors reported values of α = 3.72 ± 0.55, 4.20 ± 0.27, and 5.66 ± 0.56 for nanoemulsions with average oil droplet radius  = 89, 47, and 32 nm, respectively. In the limiting case of small scatterers such that *x*_s_ ≪ 1, Rayleigh scattering prevails and α should approach 4.^37^ However, here, α was significantly larger than 4 for  = 32 nm. The authors attributed the excessively large value of α to the sensitivity of eq 8 to β_∞_. Moreover, the authors used the so-called low-q structure factor to correct for dependent scattering effects in their calculations of the extinction coefficient. Though good agreement between numerical predictions and experimental measurements of the extinction coefficient was observed, the authors operated within the limits of single scattering for the thinnest cuvettes. Finally, Graves and Mason^13^ stated that “developing a theory that can predict the scattering properties from concentrated nanoemulsions that have significant size polydispersity would have important practical consequences.”

McClements^36^ predicted the color of emulsions using the CIE color scale through theoretical predictions of the spectral diffuse reflectance *R*_λ_ predicted by the Kubelka–Munk theory.^38^ For that purpose, the author employed the Lorenz–Mie theory to obtain the scattering efficiency factor *Q*_sca,λ_ and the asymmetry factor *g*_λ_ of a single oil droplet in water. The emulsion’s effective spectral absorption coefficient was approximated as the sum of the absorption coefficients of water and oil weighted by their respective volume fraction. The simulated emulsions had an oil volume fraction *f*_v_ of up to 10% and a droplet radius of *r*_s_ = 0.1, 1, or 10 μm. Although the author acknowledged that dependent scattering may take place at high concentrations, it was not accounted for. In fact, without empirical corrections, the predictions did not quantitatively agree with experimental measurements of the emulsion’s color.

Zhu *et al.*(39) used refractometry to successfully retrieve the unknown initial surfactant concentration *C* and oil volume fraction *f*_v_ for PDMS, hexadecane, PPMS, and squalene oil-in-water nanoemulsions having a mean droplet radius of  100 nm, an initial oil volume fraction of 0.05 < *f*_v_ < 0.35, and an initial surfactant concentration of 150 mM < *C* < 800 mM. To do so, the authors treated their nanoemulsions as an effective medium and measured the normalized effective refractive index χ(*f*_v_, *C*) = (*n*_eff_ – *n*_m_)/*n*_m_ using an Abbé refractometer loaded with approximately 0.2 mL of their low-turbidity nanoemulsions. The oil volume fraction *f*_v_ and surfactant concentration *C* were varied by incrementally diluting their nanoemulsions. Their measurements of χ(*f*_v_, *C*) were then fitted to the so-called Biot–Arago equation,^39^ and the initial volume fraction and surfactant concentration were retrieved. Moreover, their study demonstrated the value of using optical techniques to nondestructively obtain important information about nanoemulsions, especially those containing rare materials.

Finally, most studies on oil-in-water nanoemulsions used DLS to measure their droplet size distribution.^2,9,13,14^ Then, the nanoemulsions under examination must be diluted sufficiently to ensure that single and independent scattering prevails. However, it remains unclear as to what degree a given nanoemulsion must be diluted to satisfy this condition. For example, Kim *et al.*(14) diluted PDMS oil-in-water nanoemulsions to reach a volume fraction *f*_v_ = 1% in preparation for DLS measurements and obtained a droplet diameter *d*_s_ ranging from 40 to 60 nm. Qian and McClements^9^ diluted corn oil/octadecane-in-water nanoemulsions to a volume fraction *f*_v_ of around 0.05% and measured the droplet diameter *d*_s_ between 80 and 1130 nm. Meleson *et al.*(2) diluted PDMS oil-in-water nanoemulsions from oil volume fraction *f*_v_ of 20 to 0.001% to achieve concentrations below the surfactant critical micelle concentration (CMC) to ensure that the detected light scattering was only due to oil droplets and not due to micelles. They obtained droplet diameters *d*_s_ ranging from 30 to 400 nm. Such a wide range of the dilution ratio highlights the need to understand light transfer and scattering in thick nanoemulsions in order to define the proper DLS experimental procedure.

The present study aims to explain and predict the unique optical behavior and transparency of thick and concentrated nanoemulsions previously observed and described in the literature.^2^ To do so, oil-in-water nanoemulsions with oil volume fraction varying from 1 to 20% were prepared, and their spectral normal–hemispherical transmittance was measured between 400 and 800 nm. The measurements were compared with predictions from Beer–Lambert law^13,28,36^ and from solving the RTE for light transfer through nanoemulsions accounting for independent and multiple scattering. In addition, the R^2^T^2^ method accounting for dependent and multiple scattering was also used.^24,25^

## 3 Analysis

Let us consider a nanoemulsion of thickness *L* consisting of polydisperse oil droplets of size distribution *f*(*r*_s_) randomly distributed in water with overall oil volume fraction *f*_v_ and subjected to normally incident radiation, as illustrated in Figure 1. The complex index of refraction of oil droplets was denoted by *m*_s,λ_ = *n*_s,λ_ + *ik*_s,λ_, while water was assumed to be nonabsorbing with refractive index *n*_m,λ_. Here, the optical properties of PDMS (oil) and water in the spectral range between 400 and 900 nm were taken from refs (40) and (41), respectively, and are plotted in the Supporting Information.

The spectral normal–hemispherical transmittance *T*_nh,λ_ was predicted numerically by solving the RTE [Equation 1] and assuming either independent scattering or accounting for dependent scattering. Simulations assuming independent scattering used the effective scattering σ_s,λ_ and absorption κ_λ_ coefficients of the nanoemulsions given by eq 3 and the corresponding scattering phase function  for monodisperse spherical droplets. Solutions to the RTE for volume fraction *f*_v_ ranging between 1 and 20% and droplet radius *r*_s_ ranging from 20 to 60 nm were obtained using the Monte Carlo method.^42^ Here, the boundary reflectances at the air/cuvette/nanoemulsion interfaces were ignored, and *M* normally incident rays directly entered the suspension and were traced. Such a boundary condition is equivalent to experimentally using a cuvette of water as a 100% transmittance baseline correction and allows for a direct comparison between experimentally measured and numerically simulated nanoemulsion transmittance. The normal–hemispherical transmittance, reflectance, and absorptance of the nanoemulsion were computed as follows
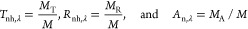
9

Here, *M*_T_, *M*_R_, and *M*_A_ are the numbers of rays transmitted in the forward hemisphere, reflected or backscattered in the backward hemisphere, and absorbed by the nanoemulsion, respectively. In addition, the total number of rays *M* is conserved so that M = *M*_T_ + *M*_R_ + *M*_A_, *i.e.*, *T*_nh,λ_ + *R*_nh,λ_ + *A*_n,λ_ = 1. A similar approach was followed for polydisperse droplets by using the expressions for κ_λ_, σ_s,λ_, and Φ_λ_ given by eqs 4 and 5.^43^

Simulations accounting for dependent scattering effects were performed using the R^2^T^2^ method described in refs (23–25). In brief, a large number *N* = 300, 500, or 900 of spherical particle ensembles of radius *R*_c_ = 5*r*_s_ were generated by cropping them from the center of *N* cubes of height 4*R*_c_ filled with randomly distributed oil droplets of size distribution *f*(*r*_s_) corresponding to volume fraction *f*_v_ varying between 1 and 20%. Here, the droplet ensembles were generated using a random packing algorithm for nonoverlapping hard spheres.^23,25^ It should be noted that although no correlation function was explicitly used in the generation of the nanoemulsions, the droplet positions were strongly correlated implicitly due to the restriction of not allowing droplets to overlap. The ensemble-averaged incoherent extinction coefficient , the incoherent scattering albedo ω_λ,*i*_^ic^, and the scattering phase function  of each particle ensemble “*i*” (1 ≤ *i* ≤ *N*) were computed using the numerically exact T-matrix method, as previously described. Then, the normal–hemispherical transmittance *T*_nh,λ_, reflectance *R*_nh,λ_, and absorptance *A*_n,λ_ of nanoemulsion slabs of thickness *L* = 10 mm were determined by using the Monte Carlo ray tracing method.^23,26^ It should be noted that the R^2^T^2^ method traces the initial planar wave originating from within the nanoemulsion, *i.e.*, it does not account for boundary reflections between the air/cuvette/nanoemulsion interfaces, as discussed in detail in ref (23). Such a boundary condition can be directly compared to experimental measurements using an appropriate baseline transmittance correction, as previously described. This is justified because (i) the incident light is mostly transmitted directly and unscattered, (ii) droplets backscatter the incident light mostly between 0° and 30°, and (iii) total internal reflection at the air/cuvette/water system occurs only for incidence angles beyond 50° (see Table S1 and Figure S5 in the Supporting Information).

Finally, in order to achieve numerical convergence, a total of *M* = 10^4^ rays were necessary when accounting for dependent scattering and *M* = 10^6^ rays when assuming independent scattering. Such a difference in the number of statistically significant rays was due to the use of the peel-off technique^44,45^ when accounting for dependent scattering. Here, the probability that a photon leaves the system is determined and added to the reflectance or transmittance depending on its direction. Although each ray tracing is computationally more expensive with this technique, a better convergence and lower noise can be reached with fewer rays.

## 4 Materials and Methods

### 4.1 Sample Preparation

**Figure 2:**
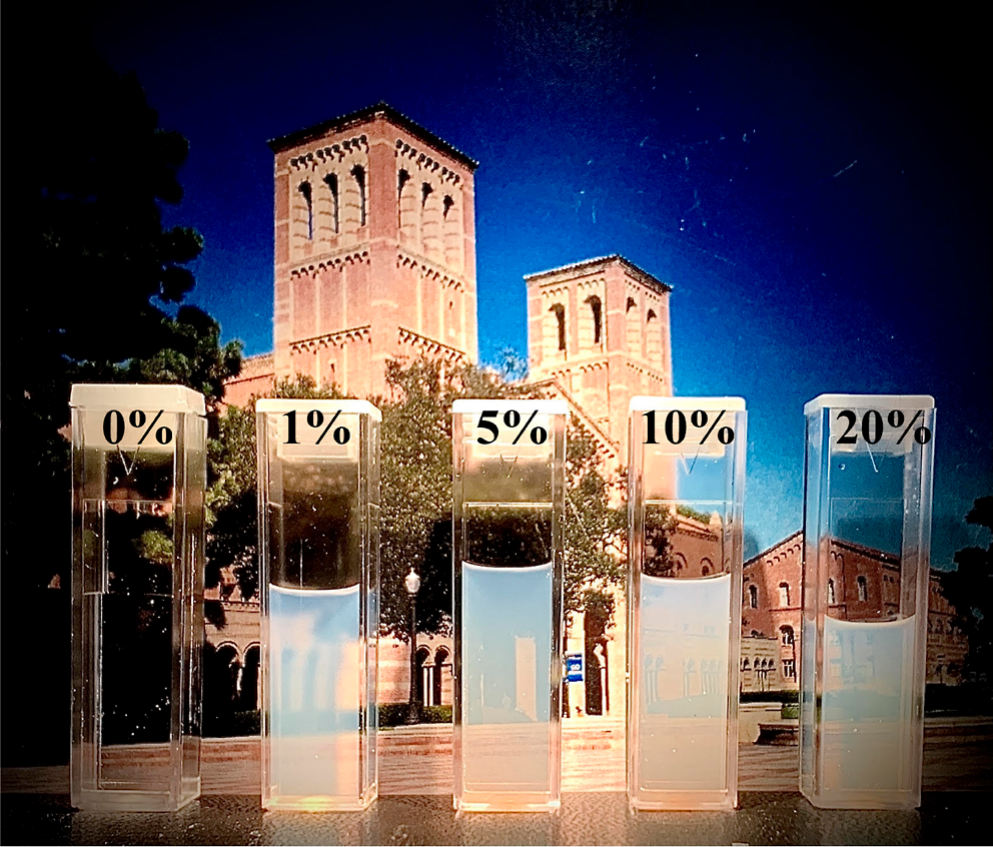
Photograph of PDMS oil-in-water nanoemulsions with PDMS volume fraction *f*_v_ ranging from 0% (DI water) to 20% in polystyrene cuvettes of path length *L* = 10 mm.

Oil-in-water nanoemulsions were synthesized based on the two-step procedure proposed by Meleson *et al.*(2) Here, commercially available PDMS silicone oil (Sigma-Aldrich) with kinematic viscosity ν = 5 cSt and aqueous sodium dodecyl sulfate (SDS) solution (Fisher BioReagents) with 0.69 M concentration were used as received. First, 20 mL of PDMS oil was dispersed in 80 mL of aqueous SDS solution with a Branson Digital Sonifier probe sonicator operated for 30 min at a power of 240 W. The temperature was maintained near room temperature by immersing the beaker in an ice bath. This step resulted in a hazy PDMS oil-in-water emulsion with PDMS volume fraction *f*_v_ = 20%. Second, the extreme shear stress necessary to rupture the emulsion’s microscale oil droplets into nanoscale droplets was applied using a Microfluidics M-110P microfluidizer. The emulsion was passed through the microfluidizer three times at a system pressure of 20,000 psi. This second step led to a semitransparent PDMS oil-in-water nanoemulsion. Finally, the produced nanoemulsion was diluted with deionized water to produce PDMS oil-in-water nanoemulsions with volume fraction *f*_v_ ranging from 1 to 20%, as illustrated in Figure 2.

### 4.2 Sample Characterization

#### 4.2.1 Droplet Size Characterization

DLS measurements were performed using a Malvern DLS/Zetasizer instrument at the scattering angle θ = 173° and wavelength λ = 632 nm. For that purpose, 100 μL of the nanoemulsion with oil volume fraction *f*_v_ = 1% was added to 2 mL of DI water in a 10 mm thick polystyrene cuvette. This dilution achieved an oil volume fraction of *f*_v_ = 4.8 × 10^–4^. The corresponding SDS concentration of 0.033 mM was well below the SDS CMC of 8 mM.^2^ The size distribution *f*(*r*_s_) was retrieved as a function of PDMS droplet radius *r*_s_ after averaging over five measurements.

#### 4.2.2 Optical Characterization

The nanoemulsions with PDMS oil volume fraction *f*_v_ ranging from 1 to 20% were placed in polystyrene cuvettes of path length *L* = 10 mm. Their normal–hemispherical transmittance *T*_nh,λ_ was measured using a double-beam ultraviolet–visible (UV–vis) spectrophotometer (iS50, Fisher Thermo Scientific, USA) equipped with an integrating sphere (EVO220, Fisher Thermo Scientific, USA). Data were collected in the spectral range between 400 and 900 nm in 1 nm spectral increments. The baseline transmittance measurement was performed with a polystyrene cuvette containing only DI water to correct for any reflection at the air/cuvette and cuvette/water interfaces. Due to the strong direct transmittance and narrow-angle scattering nature of nanoemulsions (see Table S1 and Figure S5 in the Supporting Information), this choice of baseline is representative of the system and enables us to directly compare the spectral transmittance measurements with the numerical predictions by assuming independent scattering or accounting for dependent scattering, using the boundary conditions previously described.

Measurements of the nanoemulsions’ normal–hemispherical reflectance *R*_nh,λ_ were performed using an eight degree wedge and the above-mentioned integrating sphere. Here, a Spectralon standard mirror of known spectral reflectance was taken as the baseline measurements. However, it should be noted that the normal–hemispherical reflectance of the nanoemulsions was nearly independent of volume fraction, as previously observed in suspensions of silica nanoparticles and microalgae,^46^ and was dominated by the reflectance at the cuvette/air interface (see the Supporting Information). Note that these reflectance measurements could not be directly compared with predictions of the normal–hemispherical reflectance since the latter ignored the presence of the cuvette and any reflection at the air/cuvette interface.

## 5 Results and Discussion

### 5.1 Numerical Simulations

#### 5.1.1 Effect of Oil Volume Fraction *f*_*v*_

**Figure 3:**
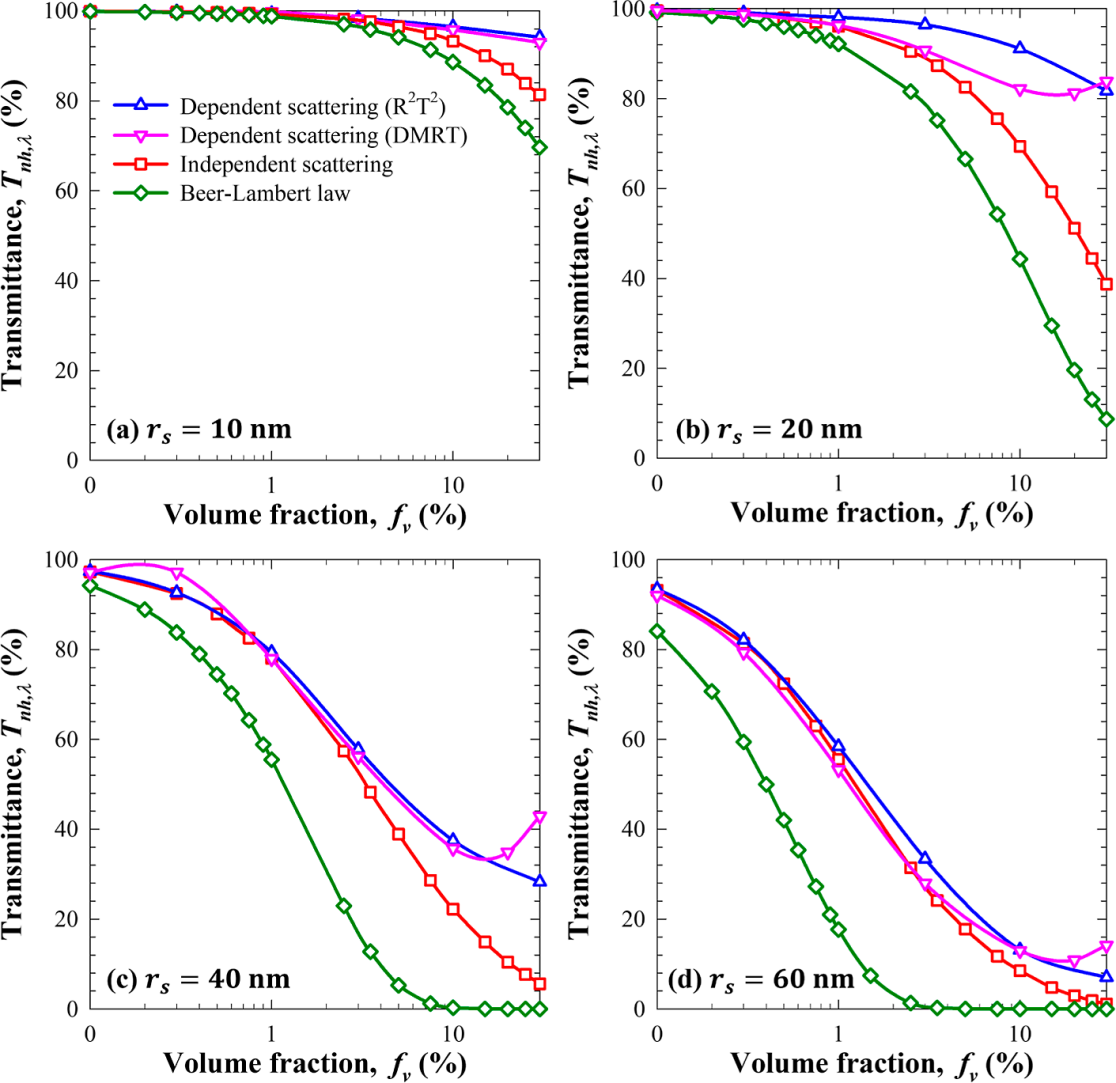
Normal–hemispherical transmittance *T*_nh,λ_ at wavelength λ = 600 nm predicted as a function of oil volume fraction *f*_v_ by Beer–Lambert law or by solving the RTE assuming independent scattering or accounting for dependent scattering using the DMRT and R^2^T^2^ methods. The nanoemulsions had thickness *L* = 10 mm and consisted of monodisperse PDMS droplets in water with radius (a) *r*_s_ = 10 nm, (b) *r*_s_ = 20 nm, (c) *r*_s_ = 40 nm, and (d) *r*_s_ = 60 nm.

Figure 3 plots predictions of the normal–hemispherical transmittance *T*_nh,λ_ of nanoemulsions at wavelength λ = 600 nm as a function of PDMS droplet volume fraction *f*_v_ for monodisperse droplet radius (a) *r*_s_ = 10 nm, (b) *r*_s_ = 20 nm, (c) *r*_s_ = 40 nm, and (d) *r*_s_ = 60 nm and thickness *L* = 10 mm. Here, the wavelength of 600 nm was chosen arbitrarily such that *m*_s,600_ = 1.41 + *i*9.32 × 10^–7^ and *n*_m,600_ = 1.33. Predictions include (i) Beer–Lambert law given by eq 2 and (ii) solutions to the RTE using the Monte Carlo method assuming independent scattering, (iii) using the DMRT method, or (iv) using the R^2^T^2^ method accounting for dependent scattering. First, Figure 3 indicates that the transmittance *T*_nh,600_ decreased with increasing volume fraction *f*_v_ and/or droplet radius *r*_s_. Predictions assuming independent scattering were in good agreement with those accounting for dependent scattering using the R^2^T^2^ method for small volume fractions *f*_v_ ≤ 1% for all values of droplet radius *r*_s_ considered in a 10 mm thick nanoemulsion. Alternatively, the DMRT method predicted that dependent scattering prevailed for volume fractions *f*_v_ > 3%. For small particle radius of *r*_s_ = 10 nm, simulations accounting for dependent scattering using the DMRT and R^2^T^2^ methods were in good agreement. However, for larger droplet radius *r*_s_ > 10 nm and volume fraction *f*_v_ > 10%, the DMRT method predicted large increases in transmittance, which was observed experimentally at larger oil volume fractions. It is interesting to note that spatial correlations become increasingly important as the droplet volume fraction increases. Likewise, increasing the droplet volume fraction also increases contributions from near-field scattering effects.^47^ Thus, the DMRT method based on the structure factor gave good predictions of the transmittance for volume fractions *f*_v_ ranging from 1 to 10%, but it failed for larger volume fractions^47^ due to the increasing importance of near-field effects. Similar findings and conclusions were reported by Yalçin *et al*.^23^ Finally, note also that in the predictions of the R^2^T^2^ method, particles were assumed to be nonoverlapping randomly distributed in the nanoemulsions for a given oil volume fraction while solving rigorously for EM wave scattering by these droplets. The predictions agreed well with experimental data for various wavelengths and oil volume fractions. Additionally, the maximum volume fraction up to which scattering could be assumed to be independent increased with increasing oil droplet radius. This can be attributed to the fact that, for a given volume fraction *f*_v_, the number of droplets per unit volume *N*_T_ decreased as the droplet size increased so that the minimum distance separating them increased beyond 5λ when independent scattering prevails.^30^

Figure 3 also indicates that the commonly used Beer–Lambert law severely underestimated the transmittance of nanoemulsions in a standard 10 mm cuvette even at a low volume fraction for all droplet radii *r*_s_ considered. Deviations between predictions by Beer–Lambert law and numerical solutions of the RTE assuming independent scattering increased with increasing oil volume fraction and were due solely to multiple scattering which is ignored by Beer–Lambert law. In practice, these results establish that for DLS characterization, nanoemulsions should be diluted to oil volume fraction *f*_v_ ≪ 0.1% and, the larger the oil droplets are, the more diluted they should be so as to minimize multiple scattering. More importantly, results like those plotted in Figure 3 may be used to determine the validity of single independent scattering, multiple independent scattering, and dependent and far-field without or with near-field scattering assumptions for given droplet radius and nanoemulsion thickness. For example, it may be deduced that for a nanoemulsion in a 10 mm path length cuvette, near-field effects become important for *f*_v_ > 10%, *r*_s_ ≥ 20 nm, at wavelength λ = 600 nm.

**Figure 4:**
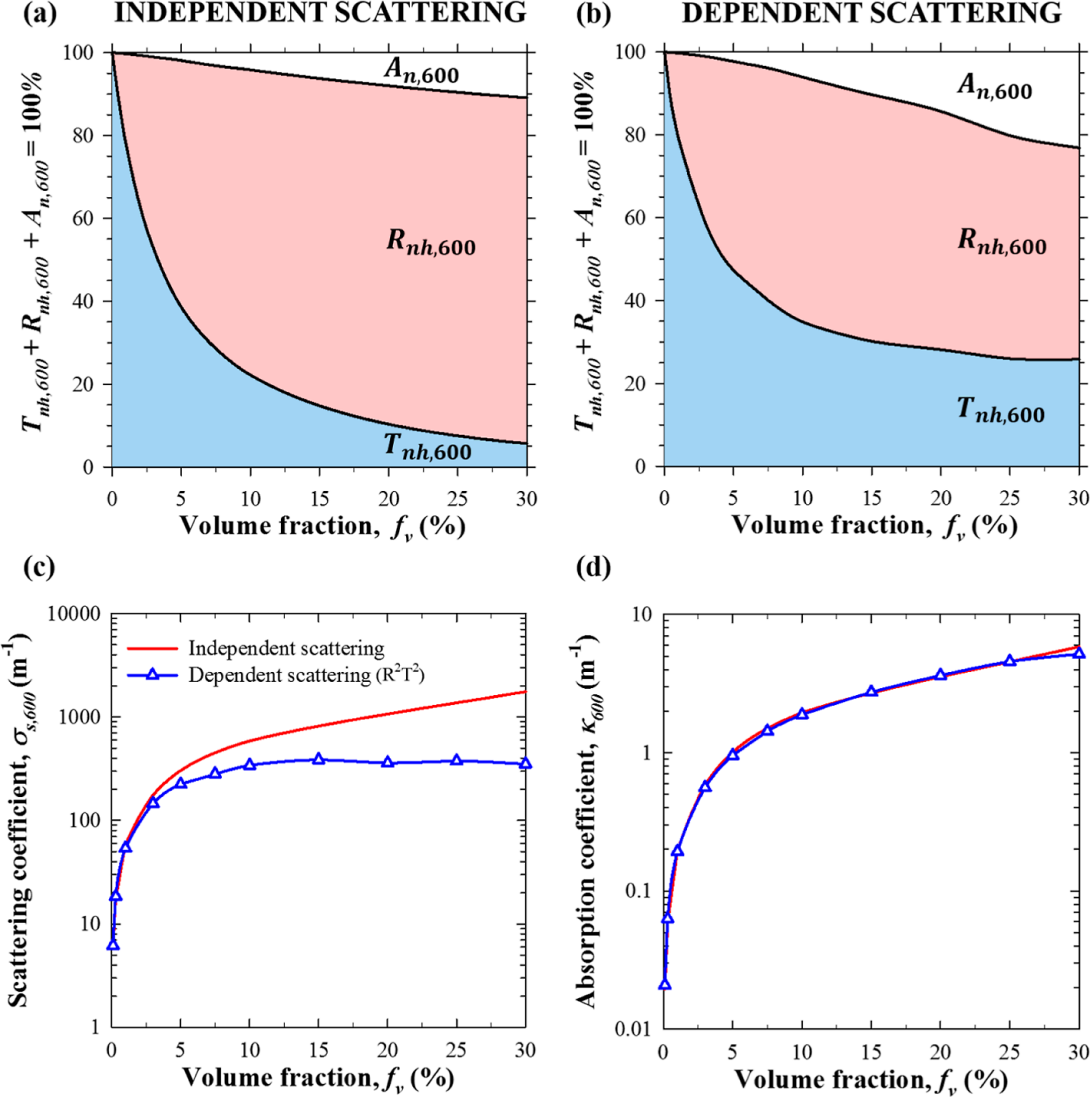
(a,b) Normal–hemispherical transmittance *T*_nh,λ_, reflectance *R*_nh,λ_, and absorptance *A*_n,λ_ at wavelength λ = 600 nm as functions of oil volume fraction *f*_v_ predicted by solving the RTE (a) assuming independent scattering or (b) accounting for dependent scattering using the R^2^T^2^ method. The nanoemulsions were 10 mm thick with monodisperse PDMS droplets of radius *r*_s_ = 40 nm. Corresponding (c) scattering σ_s,600_ (or σ_s,600_^ic^) and (d) absorption κ_600_ coefficients.

Figure 4a,b) plots the normal–hemispherical transmittance *T*_nh,λ_, reflectance *R*_nh,λ_, and absorptance *A*_n,λ_ at wavelength λ = 600 nm for a 10 mm thick nanoemulsions with monodisperse droplets of radius *r*_s_ = 40 nm as functions of PDMS oil volume fraction *f*_v_ (a) assuming independent scattering and (b) accounting for dependent scattering. Here, predictions accounting for or ignoring dependent scattering only coincided at low volume fractions *f*_v_ ≤ 1%. In fact, the normal–hemispherical transmittance *T*_nh,600_ obtained assuming independent scattering decreased sharply with increasing volume fraction *f*_v_, falling below 10% for *f*_v_ ≥ 20%. By contrast, *T*_nh,600_ reached a plateau for *f*_v_ ≥ 20% when *T*_nh,600_ ≃ 25%. The absorptance *A*_n,600_ for oil volume fractions *f*_v_ > 5% was larger when dependent scattering was accounted for, despite the fact that PDMS was weakly absorbing (*k*_s,600_ = 9.32 × 10^–7^) and water was transparent. In addition, the normal–hemispherical reflectance *R*_nh,600_ continuously increased with increasing volume fraction *f*_v_ when assuming independent scattering. It was smaller when accounting for dependent scattering and reached a maximum around *f*_v_ ≈ 10%. The above observations for *T*_nh,λ_, *R*_nh,λ_, and *A*_n,λ_ can be attributed to the fact that dependent scattering effects became increasingly important as the volume fraction *f*_v_ increased. Then, the incoherent scattering coefficient σ_s,600_^ic^ fell significantly below σ_s,600_ predicted by assuming independent scattering, as illustrated in Figure 4c for the results presented in Figure 4a,b. By contrast, the absorption coefficient κ_600_ increased with increasing *f*_v_ but was the same regardless of whether dependent scattering was accounted for, as demonstrated in Figure 4d. As a result, the photon mean free path (∼1/β_λ_) in the nanoemulsion was larger when dependent scattering prevailed. Thus, light penetrated deeper into the nanoemulsion and increased the probability of absorption by the PDMS oil droplets. Hence, the amount of backscattered light and the reflectance *R*_nh,λ_ decreased and the absorptance *A*_n,λ_ increased. By contrast, assuming independent scattering overestimated the scattering coefficient σ_s,600_ [Figure 4c] and reduced the photon mean free path in the nanoemulsion. Consequently, light was strongly backscattered near the boundary of the nanoemulsion and did not travel very deep in the nanoemulsion, which decreased the likelihood of absorption by oil droplets.

#### 5.1.2 Effect of Droplet Radius *r*_s_

**Figure 5:**
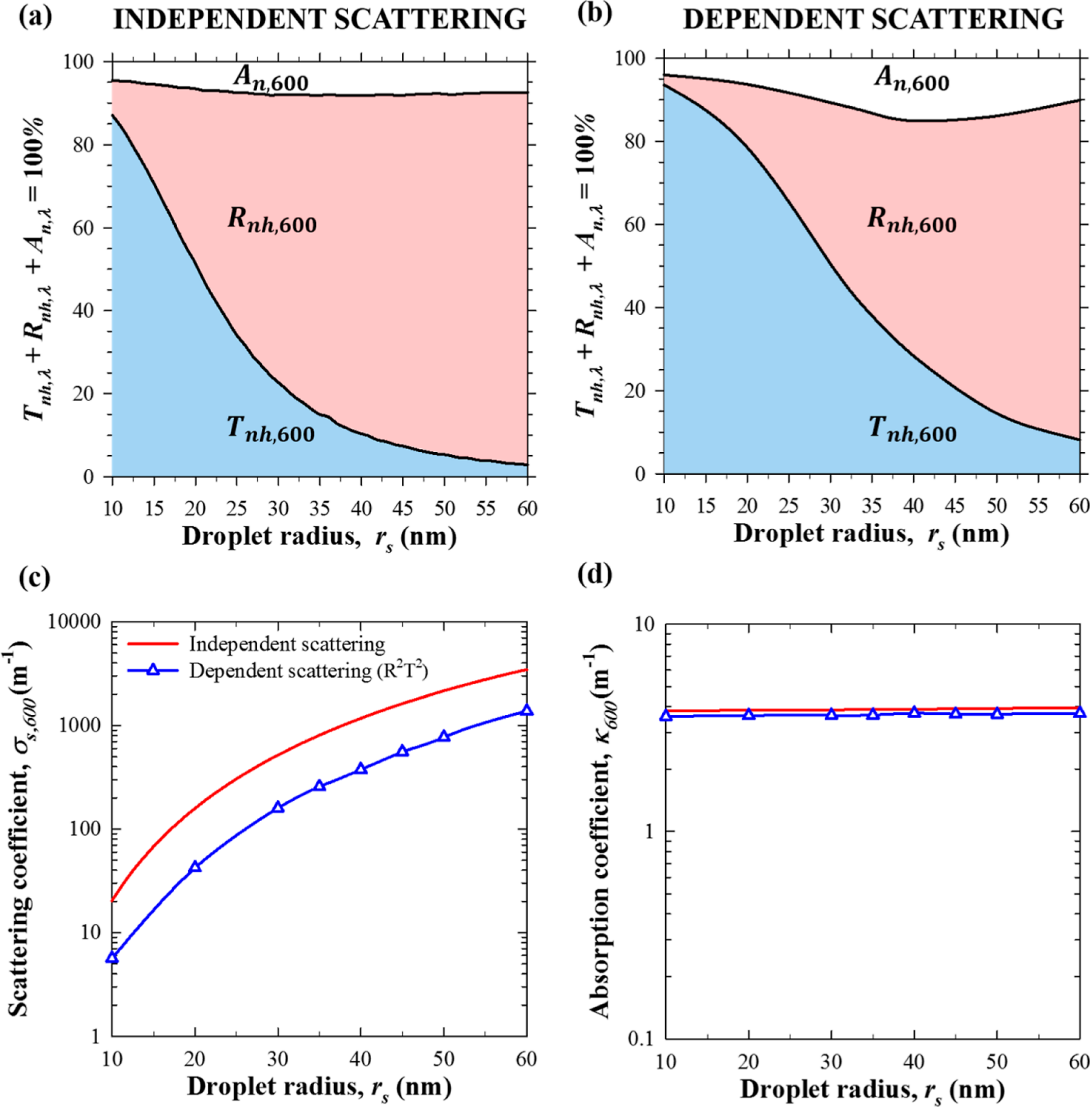
(a,b) Normal–hemispherical transmittance *T*_nh,λ_, reflectance *R*_nh,λ_, and absorptance *A*_n,λ_ at wavelength λ = 600 nm as functions of droplet radius *r*_s_ predicted by solving the RTE (a) assuming independent scattering or (b) accounting for dependent scattering using the R^2^T^2^ method. The nanoemulsions had a thickness of *L* = 10 mm and monodisperse droplets with a volume fraction of *f*_v_ = 20%. Corresponding (c) scattering σ_s,600_ (or σ_s,600_^ic^) and (d) absorption κ_600_ coefficients.

Figure 5a,b compares the normal–hemispherical transmittance *T*_nh,600_, reflectance *R*_nh,600_, and absorptance *A*_n,600_ of nanoemulsions with monodisperse PDMS oil droplets as functions of droplet radius *r*_s_ for *f*_v_ = 20% and *L* = 10 mm (a) assuming independent scattering and (b) accounting for dependent scattering. Here also, the predictions of the nanoemulsion’s transmittance *T*_nh,600_ and absorptance *A*_n,600_ were larger when accounting for dependent scattering than when assuming independent scattering for any droplet radius considered. This can be attributed to the fact that, for *f*_v_ = 20%, σ_s,600_^ic^ was three times smaller than σ_s,600_ predicted by assuming independent scattering for all droplet radii considered [see Figure 5c]. The smaller scattering coefficient was due to the oil droplets behaving as sources of evanescent spherical waves when dependent scattering prevails.^47−49^ Then, the wave incident on a droplet was the superposition of not only to the initial incident planar wave but also of the spherical waves scattered by the nearby droplets, which may interfere constructively^48^ and reduce the scattering coefficient.^49^ By contrast, the absorption coefficient κ_600_ was independent of droplet radius for a given oil volume fraction *f*_v_ [see Figure 5d] and remained unchanged whether dependent scattering was accounted for. Thus, dependent scattering resulted in deeper light penetration in the nanoemulsion, leading to reduced reflectance as well as increased transmittance and absorptance compared with situations when independent scattering was assumed, as previously discussed.

### 5.2 Experimental Measurements

#### 5.2.1 Nanoemulsion Characterization

**Figure 6:**
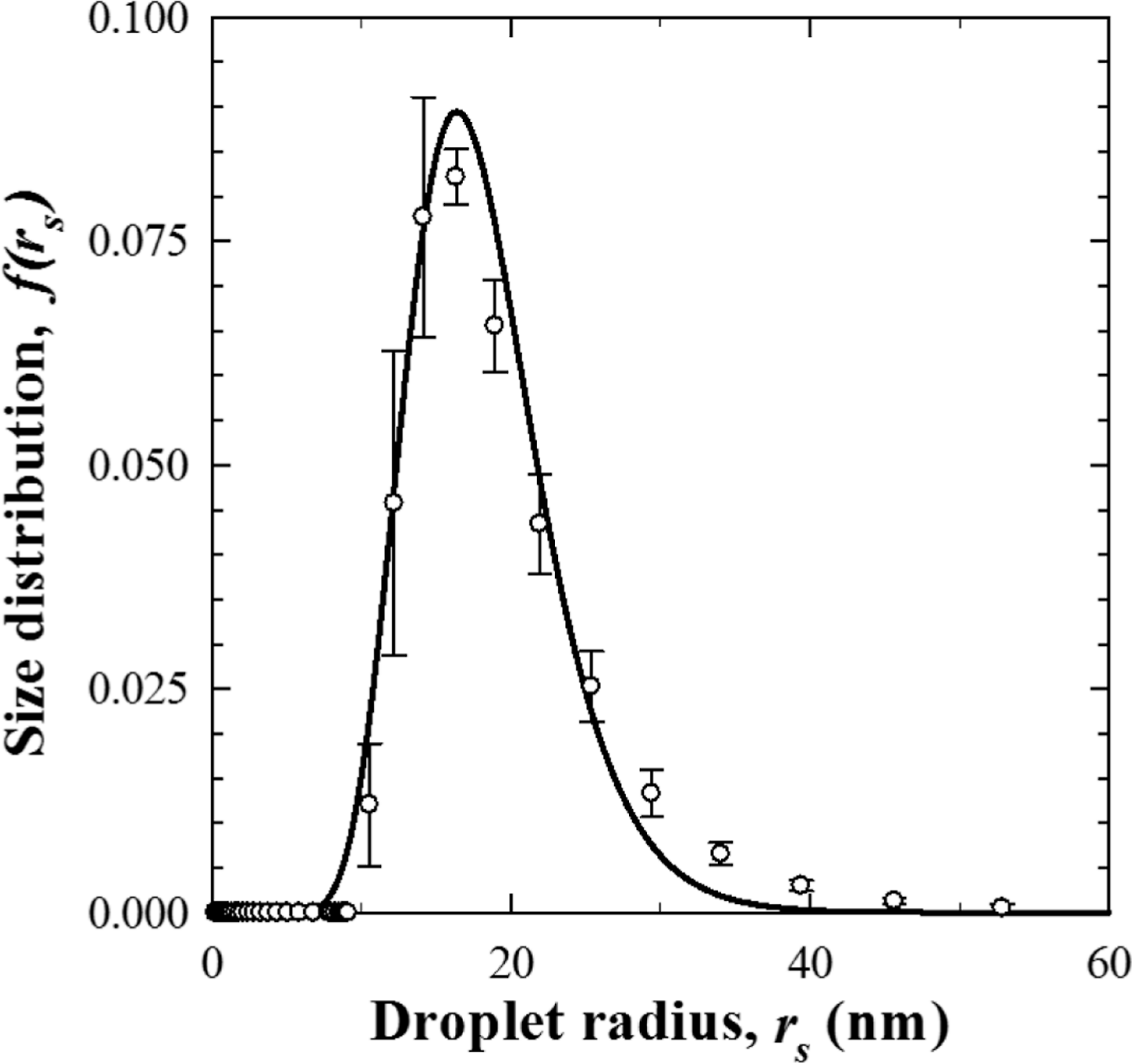
PDMS oil droplet size distribution *f*(*r*_s_) measured by DLS for the synthesized nanoemulsion along with the log–normal fit. The error bars correspond to 95% confidence interval.

Figure 6 plots the size distribution *f*(*r*_s_) of the PDMS oil droplets in the nanoemulsion obtained from DLS measurements averaged over 5 different samples of a highly diluted solution, as previously described. The DLS measurements fitted approximately to a log–normal distribution with mean μ = 2.865 and standard deviation σ = 0.263, while the peak radius was *r*_s, max_ = 16.3 nm. Repeated DLS measurements over time showed that the droplet size distribution remained nearly unchanged over the span of 1 month, indicating that the nanoemulsions were shelf stable (see the Supporting Information). These observations confirm the absence of droplet coalescence, Ostwald ripening,^10^ and phase separation for at least the duration of our experiments.

#### 5.2.2 Optical Characterization

**Figure 7:**
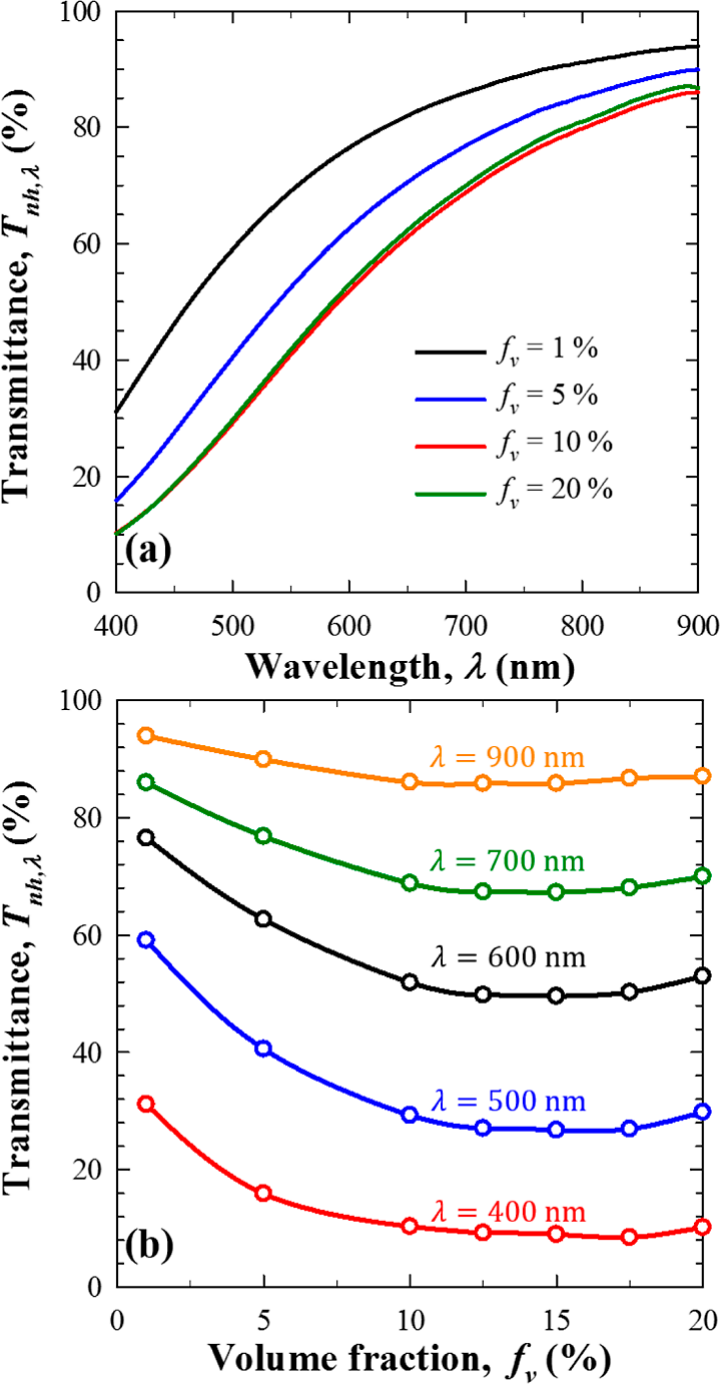
Experimental measurements of the spectral normal–hemispherical transmittance *T*_nh,λ_ of PDMS oil-in-water nanoemulsions with path length *L* = 10 mm as a function of (a) wavelength λ for volume fractions *f*_v_ ranging between 1 and 20% and (b) volume fraction *f*_v_ for wavelengths 400, 500, 600, 700, and 900 nm.

Figure 7a plots the experimentally measured spectral normal–hemispherical transmittance *T*_nh,λ_ of the nanoemulsions in the spectral range from 400 to 900 nm for PDMS volume fractions *f*_v_ = 1, 5, 10, and 20% and cuvette path length *L* = 10 mm. It establishes that the transmittance *T*_nh,λ_ increased with increasing wavelength for any given volume fraction *f*_v_ considered. This can be attributed to strong backscattering at lower wavelengths due to Rayleigh scattering. It is also interesting to note that the normal–hemispherical transmittance *T*_nh,λ_ decreased with increasing volume fraction *f*_v_ up to 10%. Beyond this volume fraction, however, the transmittance remained nearly unchanged across the spectral window 400–900 nm. In fact, Figure 7b shows the spectral normal–hemispherical transmittance *T*_nh,λ_ of nanoemulsions of thickness *L* = 10 mm plotted as a function of PDMS volume fraction *f*_v_ ranging from 1 to 20% for wavelengths λ = 400, 500, 600, 700, and 900 nm. Interestingly, the normal–hemispherical transmittance *T*_nh,λ_ at each wavelength did not decrease monotonically with volume fraction *f*_v_ as one would expect if independent scattering prevailed. Instead, *T*_nh,λ_ first decreased with increasing volume fraction *f*_v_ up to 10%. Then, it plateaued with increasing volume fraction and even increased slightly for volume fractions *f*_v_ ≥ 15%, for all five wavelengths considered. This behavior can be attributed unequivocally to dependent scattering effects. Similar observations were made previously in the numerical results of Figure 4b and experimentally with thick and concentrated colloidal suspensions.^23^

#### 5.2.3 Comparison with Numerical Simulations

**Figure 8:**
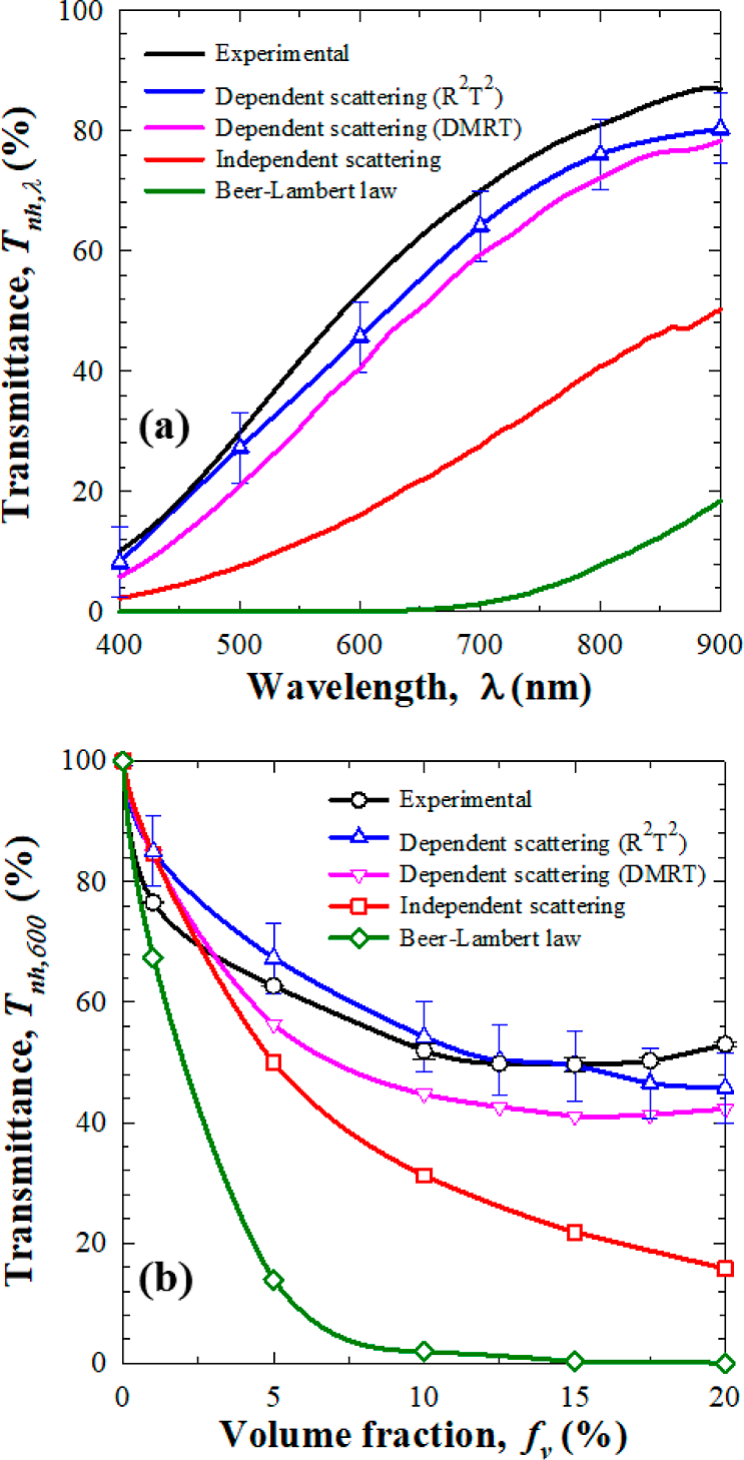
Comparison of the normal–hemispherical transmittance *T*_nh,λ_ of PDMS oil-in-water nanoemulsions of thickness *L* = 10 mm measured experimentally and numerically predicted by the Beer–Lambert law or by solving the RTE assuming independent scattering or accounting for dependent scattering using the DMRT and R^2^T^2^ methods as a function of (a) wavelength λ for *f*_v_ = 20% and (b) oil volume fraction *f*_v_ for λ = 600 nm.

Figure 8a plots the experimentally measured spectral normal hemispherical transmittance *T*_nh,λ_ between 400 and 900 nm for volume fraction *f*_v_ = 20%. It also plots numerical predictions of *T*_nh,λ_ assuming either independent scattering or accounting for dependent scattering using the DMRT and R^2^T^2^ methods. The simulations were performed using the oil droplet log–normal size distribution *f*(*r*_s_) retrieved from DLS measurements (Figure 6). Excellent agreement between numerical predictions accounting for dependent scattering and experimental measurements of *T*_nh,λ_ was observed across the spectral window. Similar results were obtained for other oil volume fractions. In fact, Figure 8b compares the experimental measurements of the normal–hemispherical transmittance *T*_nh,600_ with numerical predictions by Beer–Lambert law or by solving the RTE assuming either independent scattering or accounting for dependent scattering plotted as functions of oil droplet volume fraction *f*_v_ for nanoemulsions of thickness *L* = 10 mm. Here also, predictions of *T*_nh,600_ accounting for dependent scattering showed excellent agreement with experimental measurements for all volume fractions *f*_v_ examined. By contrast, either using the Beer–Lambert law assuming independent scattering or accounting for dependent scattering using the DMRT method severely underestimated the normal–hemispherical transmittance *T*_nh,λ_ for volume fractions *f*_v_ > 1%, as previously observed in numerical simulations.

These results establish that dependent scattering prevailed in thick and concentrated nanoemulsions. It also demonstrates the capability of the R^2^T^2^ method to account for dependent and multiple scattering unlike Beer–Lambert law or solving the RTE assuming independent scattering.

## 6 Conclusions

This study investigated numerically and experimentally light transfer through thick and concentrated PDMS oil-in-water nanoemulsions. Excellent agreement was observed between (i) experimental measurements for PDMS oil-in-water nanoemulsions with oil volume fraction ranging between 1 and 20% and peak droplet radius of 16 nm and (ii) numerical predictions of normal–hemispherical transmittance by the radiation transfer with reciprocal transactions (R^2^T^2^) method accounting for absorption as well as dependent and multiple scattering by polydisperse droplets. On the contrary, the Beer–Lambert law and the solution of the RTE assuming independent scattering severely underestimated the normal–hemispherical transmittance, especially for large oil volume fraction and small droplet radius. Additionally, numerical simulations of light transfer revealed that dependent scattering prevailed even for small volume fractions for any realistic oil droplet radius. These results offer guidelines in preparing nanoemulsions for DLS characterization. Finally, the experimental validation reported confirm the validity of the R^2^T^2^ method and its ability to rigorously account for dependent scattering. Thus, future studies may focus on light transfer through nanoemulsions containing deformed droplets or colloidal suspensions with particles of arbitrary shapes by coupling the R^2^T^2^ method with the DDA method as opposed to the T-matrix method. The R^2^T^2^ method can also conceptually be used to investigate polarized light transfer and speckle fluctuations inside and emerging from scattering media.

## Supporting Information Available

Optical properties of water and PDMS; experimentally measured nanoemulsion droplet size distributions over the span of 1 month; experimentally measured normal–hemispherical reflectance of nanoemulsions; and experimentally measured normal–hemispherical reflectance of silica nanoparticle colloidal suspension (ZIP)

## Nomenclature

*A*: absorptance
*d*: interparticle distance
*f*(*r*_s_): droplet size distribution
*f*_v_: oil volume fraction
*I*_λ_: spectral intensity
*k*: absorption index
*L*: cuvette thickness
*M*: number of ray bundles
*m*: complex refractive index
*m* =: *n* + *ik*
*N*: number of particle ensembles
*N*_T_: particle number density
*n*: refractive index
*Q*_abs,λ_^*M*^: spectral absorption efficiency factor
*Q*_sca,λ_^*M*^: spectral scattering efficiency factor
*R*: reflectance
*r*_s_: droplet radius
arbitrary direction
*T*: transmittance
*V*: volume
*x*_s_: size parameter
*x*_s_ =: 2π*r*_s_/λ
β_λ_: spectral extinction coefficient
θ: detector angle
κ_λ_: spectral absorption coefficient
λ: wavelength
ν: kinematic viscosity
ρ: density
σ_s,λ_: spectral scattering coefficient
scattering phase function
ω_λ_: scattering albedo
λ: a spectral variable
*m*: the surrounding medium
n: the normal
nh: normal–hemispherical
nn: normal–normal
s: the scattering droplets
ic: the incoherent scattering field
*M*: the Lorenz–Mie theory
