# Supplementary material for: Rigorous Accounting for Dependent Scattering in Thick and Concentrated Nanoemulsions
Source: J Phys Chem C Nanomater Interfaces. 2024 Apr 8;128(15):6419–30. doi: 10.1021/acs.jpcc.3c08072 (PMC11037395; doi:10.1021/acs.jpcc.3c08072)
Supplement: Supplementary file 1 — jp3c08072_si_001.zip [file jp3c08072_si_001.zip › Nanoemulsions/Nanoemulsions_supplementary_materials.pdf]

Rigorous Accounting for Dependent Scattering In Thick And Concentrated Nanoemulsions

— Supporting Information —

Ricardo Martinez[1], Abhinav Bhanawat[1], Refet Ali Yalçin[4], and Laurent Pilon[1,2,3*]

[1]Mechanical and Aerospace Engineering Department, Henry Samueli School of Engineering and Applied Science, University of California, 420 Westwood Plaza, Los Angeles, CA 90095, USA

[2]California NanoSystems Institute, University of California, Los Angeles, Los Angeles, California 90095, USA

[3]Institute of the Environment and Sustainability, University of California, Los Angeles, Los Angeles, California 90095, USA

[4]Institut Pprime, CNRS, Universitéde Poitiers, ISAE-ENSMA, F-86962 Futuroscope Chasseneuil, France

[*]Corresponding Author: pilon@seas.ucla.edu

February 11, 2024

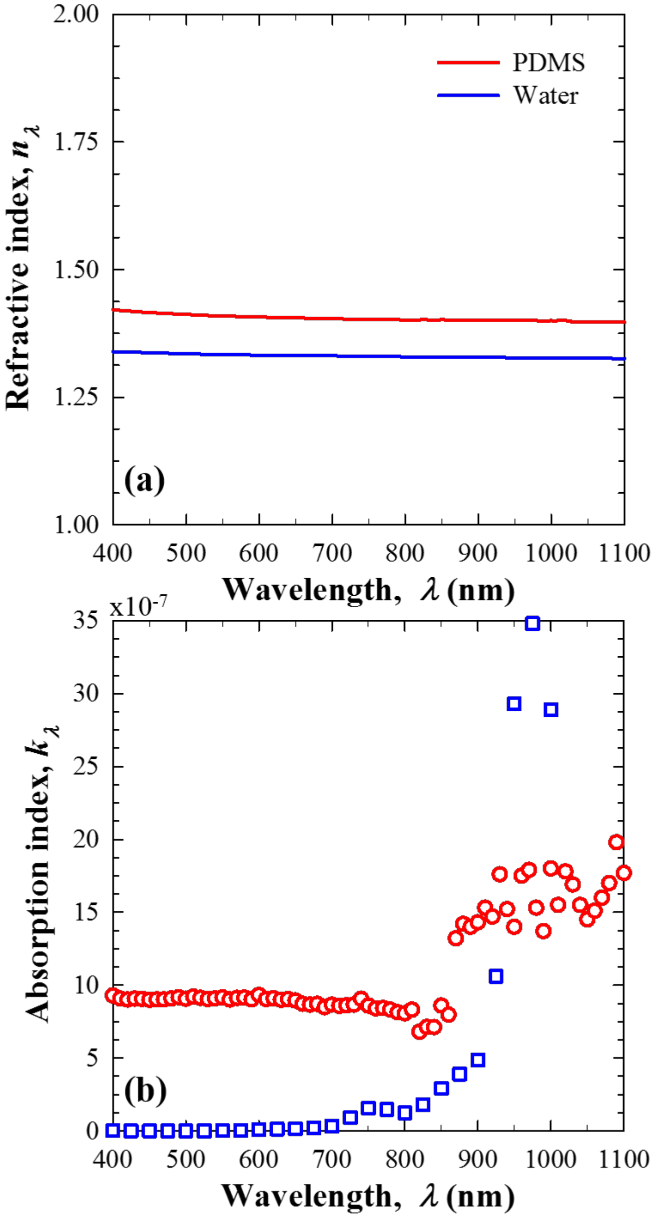

Figure S1: Spectral (a) refractive index and (b) absorption index of PDMS oil [1] and water [2] ranging between 400 and 1100 nm.

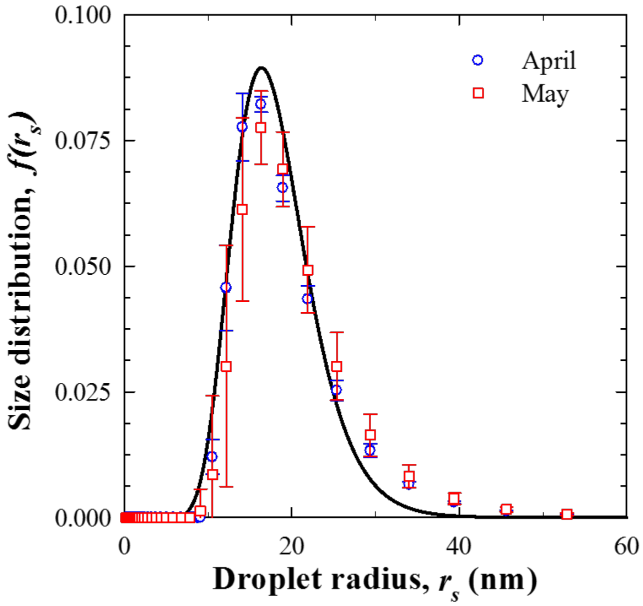

Figure S2: PDMS oil droplet size distribution $f(r)$ obtained by DLS for a PDMS oil-in-water nanoemulsion with volume fraction $f_v = 4.76\times10^{-4}$ taken on April and May 2022.

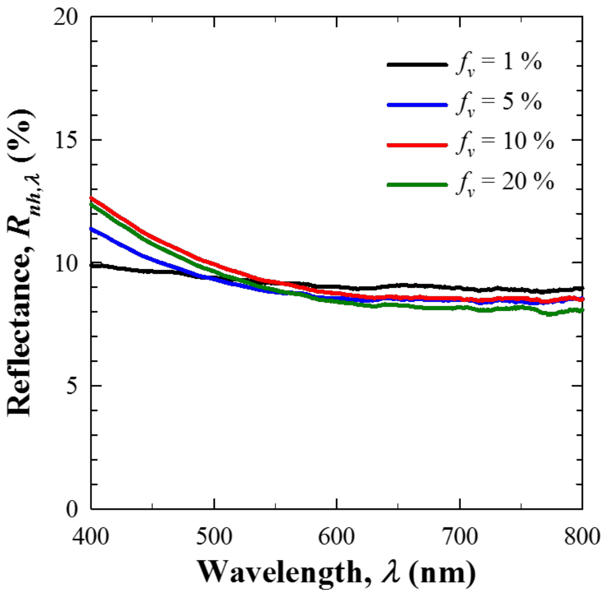

Figure S3: Experimental measurements of the spectral normal-hemispherical reflectance $R_{nh,\lambda}$ of PDMS oil-in-water nanoemulsions in polystyrene cuvettes with pathlength $L = 10$ mm as a function of wavelength λ between 400 nm and 800 nm for volume fraction f_v ranging between 1% and 20%.

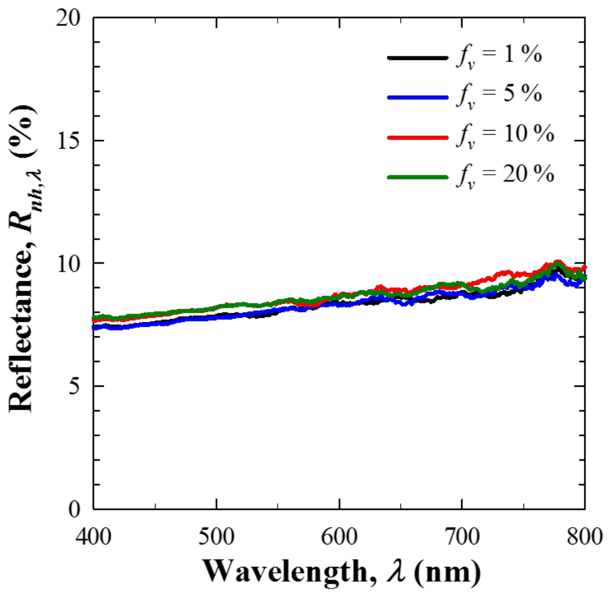

Figure S4: Experimental measurements of the spectral normal-hemispherical reflectance $R_{nh,\lambda}$ of colloidal suspension of silica nanoparticles (Nexsil 12) in polystyrene cuvettes with pathlength $L = 10$ mm as a function of wavelength λ between 400 nm and 800 nm for volume fraction f_v ranging between 1% and 20%.

Table S1: Energy balance of PDMS oil-in-water nanoemulsion having oil droplet radius r_s = 20 nm, oil volume fraction f_v = 1% to 20%, and pathlength $L = 10$ mm at wavelength λ = 600 nm.

Volume fraction (%)	Diffuse reflectance (%)	Diffuse transmittance (%)	Direct transmittance (%)	Absorptance (%)
1	3.3	3.7	92.8	0.2
20	15.3	15.3	63.4	6

REFERENCES

[1] Zhang, X.; Qiu, J.; Li, X.; Zhao, J.; Liu, L. Complex refractive indices measurements of polymers in visible and near-infrared bands. *Appl. Optics* **2020**, *59*, 2337–2344.

[2] Hale, G. M.; Querry, M. R. Optical Constants of Water in the 200-nm to 200-μm Wavelength Region. *Appl. Optics* **1973**, *12*, 555–563.

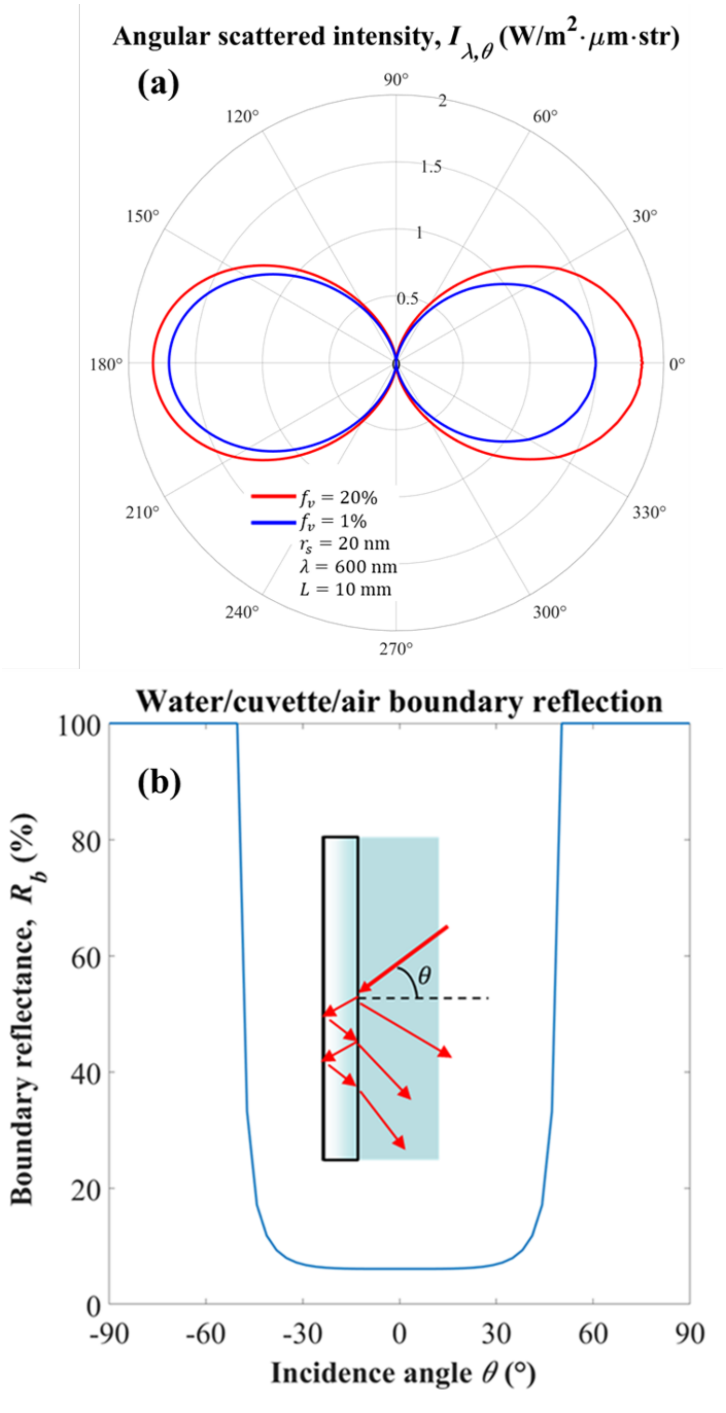

Figure S5: (a) Direction of scattered light of PDMS oil-in-water nanoemulsion having oil droplet radius $r_s = 20$ nm, oil volume fraction $f_v = 20\%$, and pathlength $L = 10$ and (b) Boundary reflectance at the water/cuvette/air interface as a function of incidence angle at wavelength $\lambda = 600$ nm.
