# Supplementary figures and images for: Rigorous Accounting for Dependent Scattering in Thick and Concentrated Nanoemulsions
Source: J Phys Chem C Nanomater Interfaces. 2024 Apr 8;128(15):6419–30. doi: 10.1021/acs.jpcc.3c08072 (PMC11037395; doi:10.1021/acs.jpcc.3c08072)

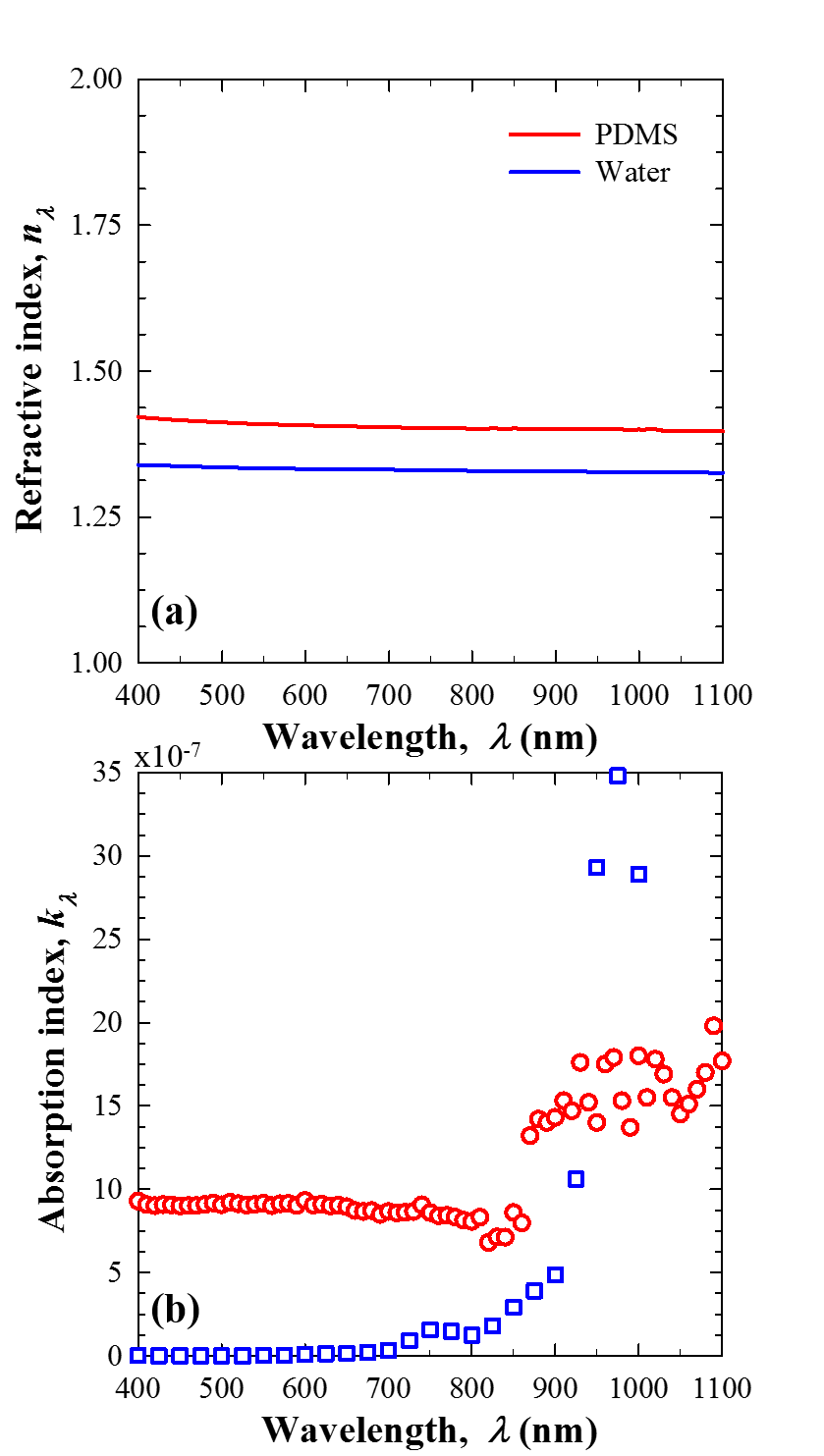

Supplement: Supplementary file 1 — jp3c08072_si_001.zip [file jp3c08072_si_001.zip › Nanoemulsions/Figures/Optical properties.png]

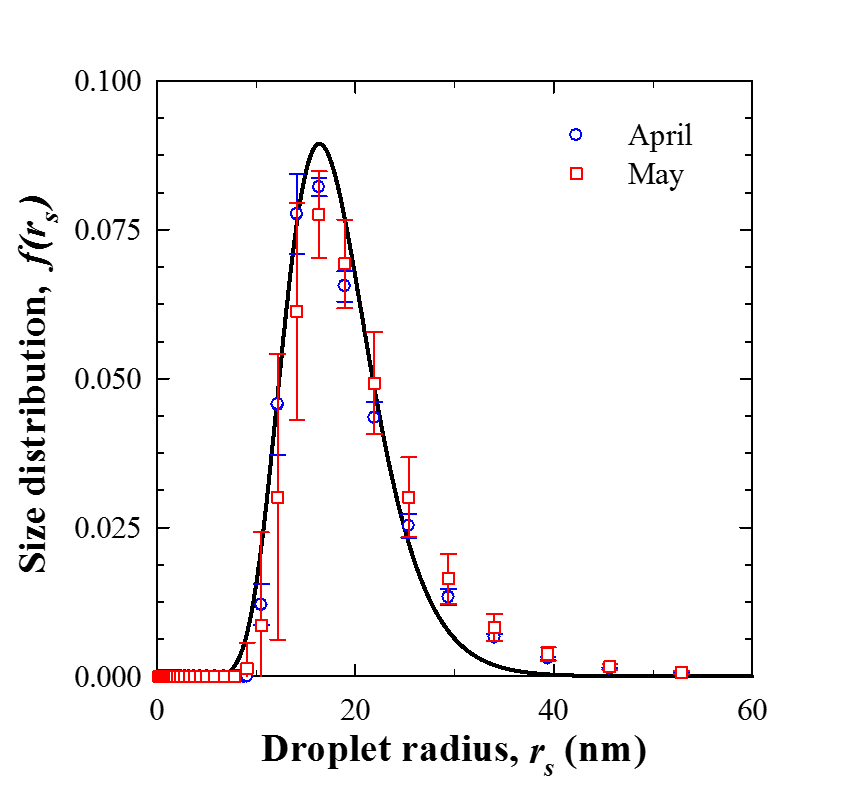

Supplement: Supplementary file 1 — jp3c08072_si_001.zip [file jp3c08072_si_001.zip › Nanoemulsions/Figures/Number_size_distributions.png]

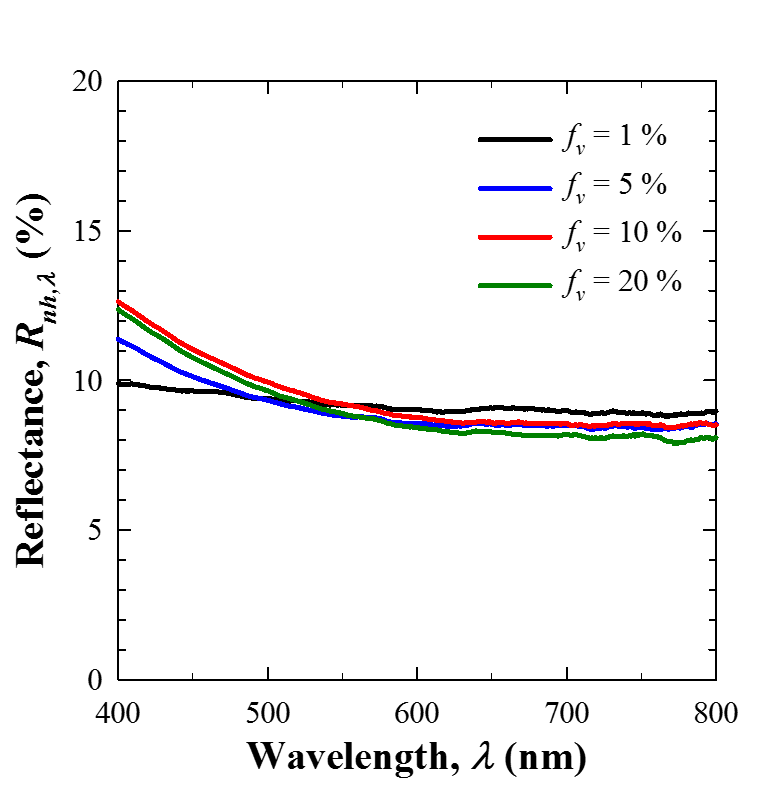

Supplement: Supplementary file 1 — jp3c08072_si_001.zip [file jp3c08072_si_001.zip › Nanoemulsions/Figures/Experimental_Reflectance.png]

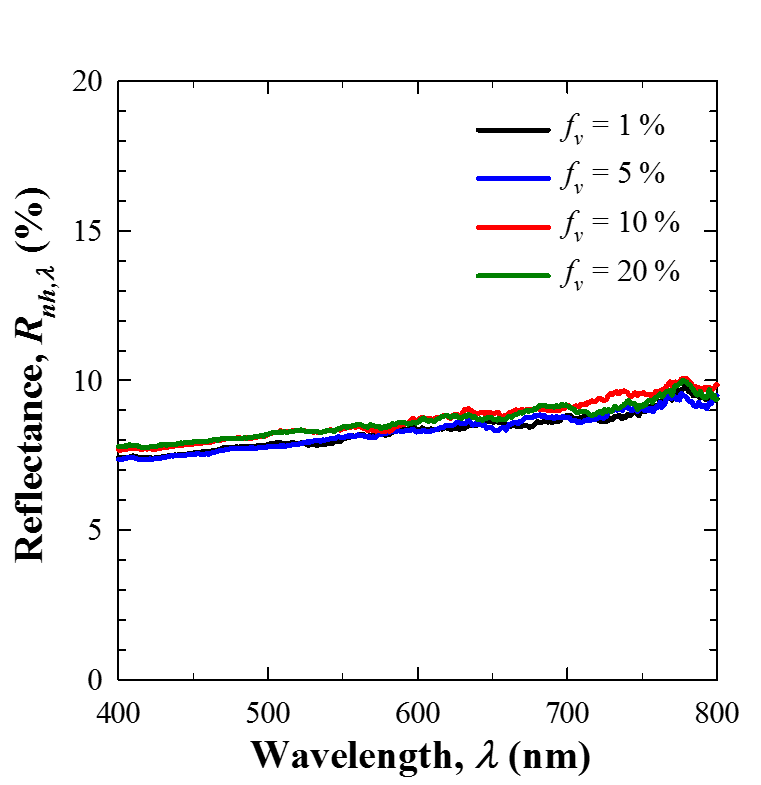

Supplement: Supplementary file 1 — jp3c08072_si_001.zip [file jp3c08072_si_001.zip › Nanoemulsions/Figures/Experimental_Reflectance_SiO2.png]

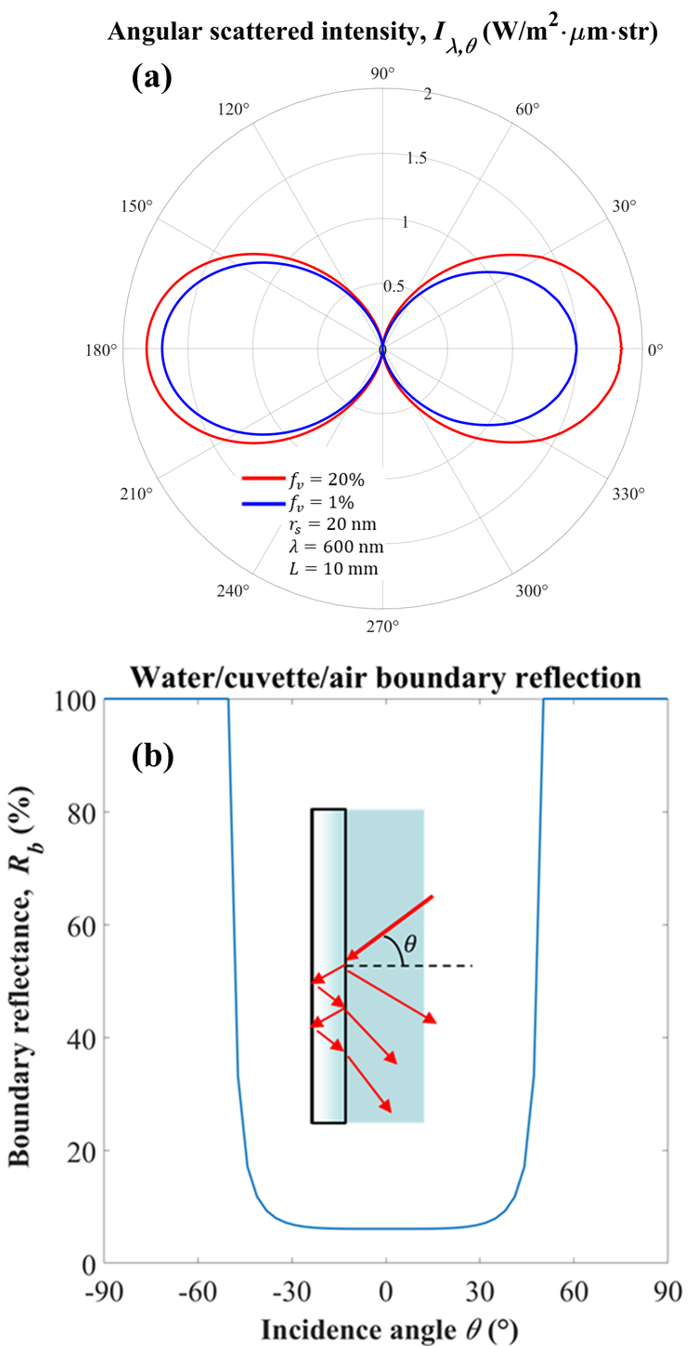

Supplement: Supplementary file 1 — jp3c08072_si_001.zip [file jp3c08072_si_001.zip › Nanoemulsions/Figures/Scattered_intensity.png]
